# ShadowCaster: Compositional Methods under the Shadow of Phylogenetic Models to Detect Horizontal Gene Transfers in Prokaryotes

**DOI:** 10.3390/genes11070756

**Published:** 2020-07-07

**Authors:** Daniela Sánchez-Soto, Guillermin Agüero-Chapin, Vinicio Armijos-Jaramillo, Yunierkis Perez-Castillo, Eduardo Tejera, Agostinho Antunes, Aminael Sánchez-Rodríguez

**Affiliations:** 1 Departamento de Ciencias Biológicas, Universidad Técnica Particular de Loja, Loja 110108, Ecuador; nela.sanchez96@gmail.com; 2 CIIMAR/CIMAR, Interdisciplinary Centre of Marine and Environmental Research, University of Porto, Terminal de Cruzeiros do Porto de Leixões, Av. General Norton de Matos s/n, 4450-208 Porto, Portugal; gchapin@ciimar.up.pt (G.A.-C.); aantunes@ciimar.up.pt (A.A.); 3 Department of Biology, Faculty of Sciences, University of Porto, Rua do Campo Alegre, 4169-007 Porto, Portugal; 4 Grupo de Bio-Quimioinformática & Carrera de Ingeniería en Biotecnología, Facultad de Ingeniería y Ciencias Agropecuarias, Universidad de Las Américas, Quito EC170125, Ecuador; vinicio.armijos@udla.edu.ec (V.A.-J.); eduardo.tejera@udla.edu.ec (E.T.); 5 Grupo de Bio-Quimioinformática & Escuela de Ciencias Físicas y Matemáticas, Universidad de Las Américas, Quito EC170125, Ecuador; yunierkis.perez@udla.edu.ec

**Keywords:** horizontal gene transfer, parametric method, implicit phylogenetic model, hybrid approach

## Abstract

Horizontal gene transfer (HGT) plays an important role for evolutionary innovations within prokaryotic communities and is a crucial event for their survival. Several computational approaches have arisen to identify HGT events in recipient genomes. However, this has been proven to be a complex task due to the generation of a great number of false positives and the prediction disagreement among the existing methods. Phylogenetic reconstruction methods turned out to be the most reliable ones, but they are not extensible to all genes/species and are computationally demanding when dealing with large datasets. In contrast, the so-called surrogate methods that use heuristic solutions either based on nucleotide composition patterns or phyletic distribution of BLAST hits can be applied easily to the genomic scale, but they fail in identifying common HGT events. Here, we present ShadowCaster, a hybrid approach that sequentially combines nucleotide composition-based predictions by support vector machines (SVMs) under the shadow of phylogenetic models independent of tree reconstruction, to improve the detection of HGT events in prokaryotes. ShadowCaster successfully predicted close and distant HGT events in both artificial and bacterial genomes. ShadowCaster detected HGT related to heavy metal resistance in the genome of *Rhodanobacter denitrificans* with higher accuracy than the most popular state-of-the-art computational approaches, encompassing most of the predicted cases made by other methods. ShadowCaster is released at the GitHub platform as an open-source software under the GPLv3 license.

## 1. Introduction

Lateral or horizontal gene transfer (HGT) plays an important role in the genome evolution and ecological innovation of prokaryotic communities. Microbial communities can be considered as complex biological systems where its individuals exchange genes by HGT events. HGTs in bacteria and archaea communities occur more frequently between closely related species than in distant lineages [1]. Some lineage-specific genes, i.e., genes found in one particular taxonomic group and that arise from close HGT events, are usually lost quickly if they result in reduced fitness. By contrast, other lineage-specific genes are retained for longer periods of time if they provide selective advantages for survival in the recipient lineage, especially when environmental conditions do not change much [2]. Rates of HGT events involving genes critical for survival, growth, and reproduction are particularly high among members of microbial communities that need a quick adaptation to complex environments such as contaminated soil or water [3]. Detecting HGT has been a major focus of attention to better understand microbial evolution. However, it has proven to be a complex and challenging task.

HGT detection tools can be divided into two main classes: parametric and phylogenetic methods. Parametric methods search for sections of a putative recipient genome that greatly differ from the mean nucleotide composition based on metrics such as oligonucleotide frequencies, guanine and cytosine (GC)content or codon usage. Genes exhibiting highly compositional fluctuations from the genomic mean are called atypical or alien genes and their origin is expected to be exogenous. Phylogenetic methods integrate information from multiple genomes and find evolutionary incongruencies while reconciling gene trees with the reference species tree [4]. Both parametric and phylogenetic methods are based on the hypothesis that acquired genes bring “perturbations” to the recipient genome, and that such perturbing signal stands out from the background noise (fluctuations in the recipient genome that arise from other scenarios rather than from HGT events), regardless the age of the event. 

To date, there is no unique method capable of detecting all the HGT events of different ages in a recipient genome because each of the methods have their own advantages and limitations. Phylogenetic methods are best at identifying ancient HGT events, providing a large number of orthologs and the generation of a reliable species tree [5,6]. Evolutionary processes other than HGT, i.e., gene duplication and differential gene loss, can also explain incongruences between genes and species trees, which hinder the performance of phylogenetic methods [7,8]. Parametric methods deal best with recent HGT events that result in noticeable perturbations to the recipient genome mean nucleotidic signature. Over time, however, gene amelioration dilutes the signal of HGT events due to the quick loss of nucleotide compositional differences which drastically reduces the detection power of parametric methods [9]. In the presence of gene amelioration, the parametric method′s predictions become inaccurate and should be validated by phylogenetic approaches to reduce both false positive and negative predictions [10,11]. Nevertheless, explicit phylogenetic methods (tree building-based) are not practical at the large scale, e.g., comprising entire gene repertoires, because they are computationally expensive and time consuming [12]. In this sense, several methods with rather implicit phylogenetic approaches have arisen. These methods are mainly based on phyletic distribution derived from BLAST searches in order to speed up atypical gene detection in multiple genomes/taxa since they bypass gene tree reconstruction, e.g., DarkHorse [13], HGTector [14] and HGT-Finder [12].

Table 1 shows the state-of-the-art of HGT detection methods that rely on different information sources and when applied to the same dataset often generate partially overlapping predictions. Discrepancies among tools might be resolved by combining parametric and phylogenetic methods. It is still unclear what could be the best way to combine different methods without increasing the false discovery rate (FDR) [4,15]. 

Here, we developed an open-source software called ShadowCaster that consists of the sequential incorporation of parametric and phylogenetic information to improve the detection of HGT events in prokaryotes. ShadowCaster firstly employs a One-class support vector machine (SVM) classifier based on two compositional features to identify putative atypical genes within the genome of the recipient species. ShadowCaster then estimates a Bayesian likelihood, inferred from an implicit phylogenetic model built by aggregating the proteomes of closely and distantly related taxa to the recipient species to construct a phylogenetic shadow. ShadowCaster finally decides, based on the Bayesian likelihood, whether the predicted atypical genes in the recipient species were vertically or horizontally acquired. To carry this out, two inheritance models—vertical and horizontal inheritance—are interrogated to see which one best explains the acquisition of an atypical gene in the recipient genome. In a pure vertical inheritance model, the number of orthologs shared by the species in the phylogenetic shadow serves as a proxy of the phylogenetic distance among species. Closely related species are supposed to share more orthologs (with higher sequence similarity) than the distantly related ones. As a result, a shared orthology probability distribution can be drawn as a curve on top of the phylogenetic shadow. In plain words, ShadowCaster detects HGT events as violations to the pure vertical inheritance model. These are cases in which an atypically conserved gene is present in a pair of species with a shared low orthology probability that is not able to explain such high sequence conservation. 

We applied ShadowCaster to predict genome-wide close and distant HGT events in artificial and bacterial genomes. The hybrid nature of ShadowCaster allowed for the prediction of new HGT events in real-world data and demonstrated its improved performance compared to pure parametric methods and to those based on phyletic frameworks. In addition, ShadowCaster predictions showed the highest agreement with those obtained by other methods.

## 2. Design and Implementation

ShadowCaster is an open-source software capable of detecting HGT events in prokaryotes by performing three major tasks: (i) atypical genes identification by One-class SVM, (ii) phylogenetic shadowing model construction, and (iii) per gene likelihood calculation expressing how likely an atypical gene has been vertically inherited. There are two main components in ShadowCaster to carry out these tasks: one parametric and one phylogenetic. The parametric component uses the genome of the query species to extract nucleotide compositional information during the task (i), while the phylogenetic component starts from the proteomes of the query and of other related species covering a diverse spectrum of phylogenetic distances, to perform tasks (ii) and (iii). 

### 2.1. Parametric Component

For the identification of atypical genes, we implemented a parametric component that combines two features, each using a particular metric to assess the compositional difference between each gene and the entire genome. Features were implemented as suggested by Becq et al. [6]. The first feature corresponded to the gene length normalized tetranucleotide (4-mers) frequencies with Chi-square as metric and the second was the codon usage with Kullback–Leibler as metric. The per-gene values of each feature were compared to the corresponding value calculated from the entire coding sequence of the genome (i.e., a concatenation of all genes in the species). 

**Figure 1:**
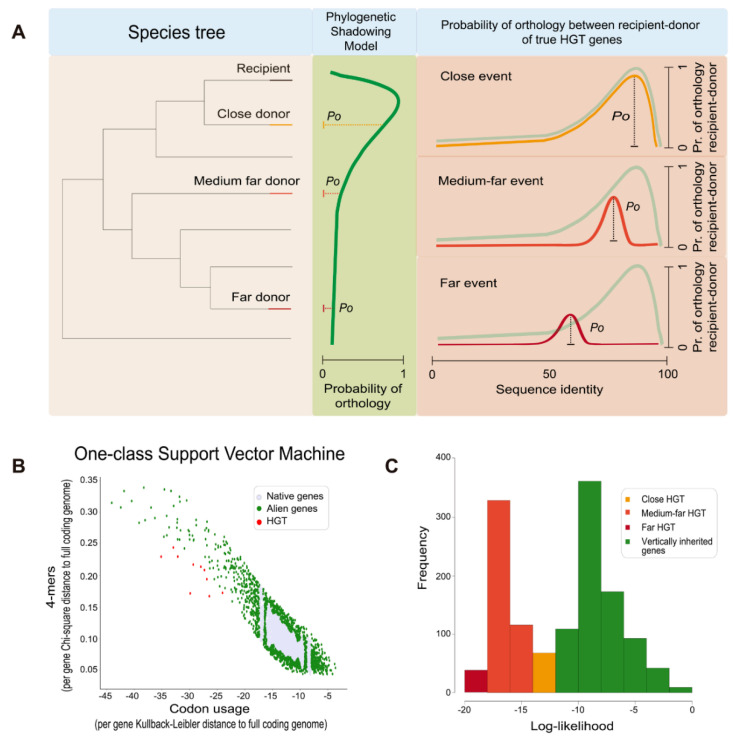
Graphical representation of the conceptual model behind ShadowCaster and of its main outputs. (**A**) Probability of true orthology between three gene pairs shared by a recipient species and three phylogenetically related donor species, respectively (species tree in the left-hand side). True orthology probability values (*P*_0_) are sampled from probability distributions according to: vertical inheritance (phylogenetic shadowing model defined by the number of orthologs sharing recipient-donor species at different phylogenetic distances, green panel); and lateral inheritance (horizonal gene transfer (HGT)) model, gradient color curves from orange to deep red represent the *P*_0_ distribution in true HGT events occurring at different phylogenetic distance along the species tree). *P*_0_ decreases in both vertical and lateral inheritances with the increase of the phylogenetic distance, however *P*_0_ distribution (curves) is different between them, especially for medium and far distances. (**B**) The distribution/separation of typical (in grey color) and atypical (in red and green color) genes achieved by the parametric component of ShadowCaster (4-mer frequency and codon usage). (**C**) Log-likelihoods for all atypical genes detected by the parametric component in a given recipient genome. Log-likelihoods and *P*_0_ are related by Equation (2).

To classify each gene as atypical based on features values, the One-class support vector machine (SVM) was used, since this method is able to perform the outlier detection in an unsupervised fashion. The goal of the One-class SVM (Figure 1B) was to separate the native genes (i.e., those with a composition that does not differ greatly from the one of the entire genomes) (gray dots in Figure 1B), from atypical genes (green and red dots in Figure 1B) by the estimation of a support distribution. This model requires the selection of a kernel function (radial basis function in our case), and the specification of the bounds of the fraction of support vectors and training errors to use as defined by the user via the “*nu* parameter” (see technical details in [21] and references therein). In our implementation, the *nu* parameter can be tuned by the user depending on the type of HGT events to detect, i.e., close, medium-far and far events (see below how the *nu* parameter affects the predictions made by ShadowCaster). Genes classified as outliers by the One-class SVM represent the group of atypical genes that constitute the output of the parametric component and one of the inputs of the phylogenetic component.

### 2.2. Phylogenetic Component

The list of atypical genes detected by the parametric component is still putative and might contain spurious results (e.g., native genes that did not arise from true HGT events). The aim of the phylogenetic component is then to create a filter capable of differentiating genes that truly arose from HGT events from those native genes that show an atypical composition but that were vertically inherited. The model behind ShadowCaster (Figure 1A) builds on the previous knowledge that the amount of true orthologous genes shared between a given species pair decreases with the phylogenetic distance that separates them [22]. In such scenario, a vertically inherited gene in a recipient species, will show a decreasing sequence identity with its orthologs in increasingly distant species. If one sees the sequence identity between two proteins as a proxy of orthology, and constructs a probability function across a species phylogenetic tree will see a shadowing shape as represented in Figure 1A (green curve). A horizontally inherited gene will in contrast disrupt the shadowing shape of the orthology probability function creating a peak around the acceptor species (see the orange, light red and deep red curves in Figure 1A). 

We took advantage of the contrasting behavior of vertically versus horizontally inherited genes in terms of orthology probability distribution across the species tree. While events of vertical and horizontal inheritance are both consistent with the shared orthology probability *P*_0_ values and distributions (green and orange curves, Figure 1A) in close-related species pairs, this is different for medium far and far species pairs. When the phylogenetic distance increases between donor and acceptor species, the shared orthology probability decreases as well as the probability of finding orthologs with high sequence identity between them. In addition, the magnitude of the decrement in shared orthology probability (*P*_0_) is different for medium and far HGT events (light red and red curves contrasted by the shadow of the green curve, Figure 1A). With such considerations, we then applied a Bayesian inference to estimate how likely is that a given gene has been vertically inherited.

When applied to our phylogenetic shadowing model, Bayes′ theorem for probability distributions can be expressed as follows (Equations (1) and (2)):

(1) f(t|y) ∝ f(y|t) f(t)

(2) P(Orthology|Identity)∝P(Identity|mean,std)P(Orthology)

where y is a vector of protein sequence identities corresponding to the best hits of an atypical sequence in each of the proteomes included in the phylogenetic shadow, and t represents the vertical inheritance model derived from comparing the full proteomes included in the phylogenetic shadow in a pairwise fashion. The distribution f(y|t) is the sampling density for y, describing the probability that an atypical sequence with an identity higher than 55% follows the vertical inheritance model; f(t) is the prior phylogenetic distribution determined by the probability of orthologs (*P*_0_) between the query species and the species in the phylogenetic shadow; finally, f(t|y) is the posterior distribution of t. Log-likelihoods for all atypical sequences detected by the parametric component in a given recipient genome were derived from Equation (2). 

To determine the number of orthologs between the species, we used OrthoMCL-pipeline (https://github.com/apetkau/orthomcl-pipeline). To derive the vertical inheritance model (P(Identity|mean,std)), the mean and standard deviation of all protein sequence identities resulted from the global alignments between each proteome pair were considered as well as the predicted atypical sequences longer than 70 amino acids. The log-likelihoods of all atypical sequences were classified in two classes with fuzzy clustering (Figure 1C). The group with lower values of likelihood represented the genes predicted as HGT events and the other the vertically inherited genes with an unusual composition.

As shown, our phylogenetic inference is based on a phylogenetic shadowing model instead of on explicitly deriving a phylogenetic tree. To construct this model, it is important to have enough species that are closely-related to the query sequence in order to detect orthology relationships, but also a correct number of species that explains sequence divergence gradually. Thus, the phylogenetic shadow is constructed by species from different phylogenetic distances based on the taxonomy of the query species. In order to best describe sequence divergence across species, our phylogenetic shadowing model is built with proteomes from species related with the query according to the following taxonomic weights: 40% of species from the same family, 20% of species from the same order, 20% of species from the same phylum, 12% of species from the same kingdom and 8% of species from another prokaryotic kingdom.

Proteomes are retrieved from the NCBI ftp by using the helper script “get_proteomes.py”. In case that there are not enough sequences to complete the percentage assigned to any of the ranks, the difference will be added to the upper taxonomic rank in order to complete with the total number of sequences to construct the phylogenetic shadow. As an example, if we consider *Escherichia coli* as the query species, its phylogenetic shadow will be composed by 40% of proteomes from Enterobacteriaceae, 20% Enterobacterales, 20% Proteobacteria, 12% Bacteria, and 8% Archaea.

ShadowCaster was mainly implemented in Python but shares functions of R and Perl too. There are minimal external dependencies (e.g., OrthoMCL [23], Blastp and EMBOSS package for running the phylogenetic component). A script to build the phylogenetic shadow is provided to help the user with the input requirements (more information is supplied in the documentation of ShadowCaster at https://shadowcaster.readthedocs.io/en/latest/).

## 3. Results and Discussion

### 3.1. Performance on Simulated Data

To determine the robustness of ShadowCaster’s approach, we conducted experiments using simulated sets from artificial gene transfers of different origins. For this purpose, an artificial genome was modeled to be the recipient sequence based on *Escherichia coli K-12 substr. MG1655* genome. Native genes were extracted from the genome using k-mers and codon usage properties, this step ensures the removal of highly atypical genes. We created three datasets with transfers from different phylogenetic distance species like *Methanocaldococcus jannaschii* (far), *Sinorhizobium meliloti* (medium-far) and *Salmonella enterica* (close). These species are also placed at different taxonomic distances to *E. coli*. *Methanocaldococcus jannaschii* (far) belongs to Archaea, order: Methanococcales, family: Methanocaldococcaceae and genus: Methanocaldococcus. *Sinorhizobium meliloti* (medium-far) is a bacterium belonging to the order: Rhizobiales, family: Rhizobiaceae and genus: Sinorhizobium. *Salmonella enterica* is a closely related bacterium to *E. coli,* both belong to the same order and family. For each set, we randomly transferred ten genes that had no orthology with the recipient sequence from the donor species previously mentioned.

**Figure 2:**
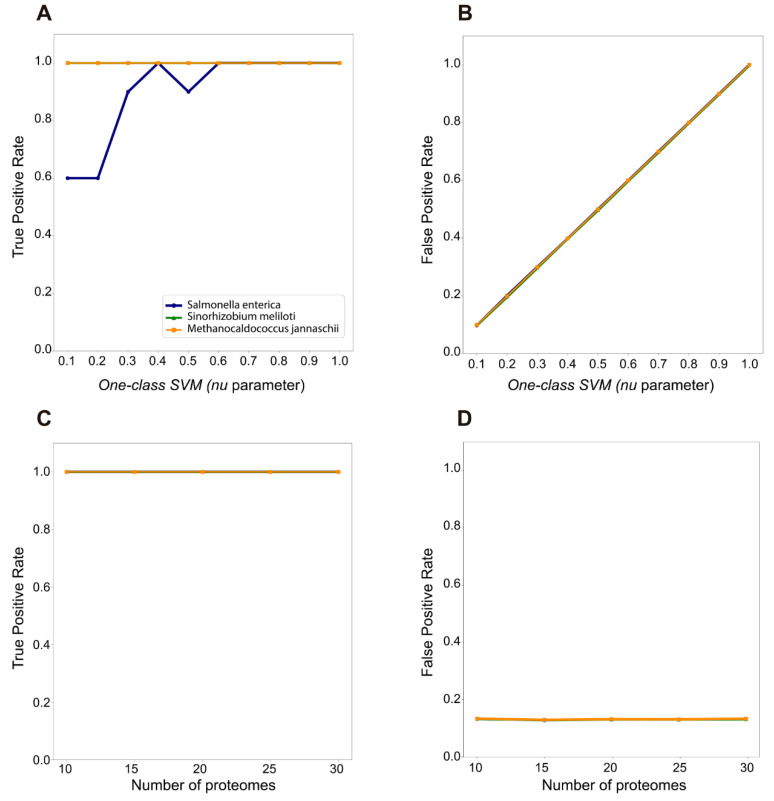
True positive (TP) and False positive (FP) rate curves for showing the performance of ShadowCaster according to the two user defined parameters: *nu* and number of proteomes. TP and FP rate curves corresponding to the *nu* parameter are shown in (**A**,**B**) while (**C**,**D**) display the same curves influenced by the number of proteomes.

We tested the effect of two parameters of ShadowCaster: *nu* parameter and the number of proteomes needed to construct the phylogenetic shadow. The *nu* parameter used in the parametric component was varied from 0.1 to 1.0 and the number of proteomes was changed from 10 to 30. The purpose of these tests is to find the optimal combination of these parameters to achieve the best performance of ShadowCaster depending on the origin of HGT events. Figure 2 shows the curves for True and False Positive Rates of the tested parameters.

The influence of the *nu* parameter was only evaluated on the HGT detection within the group of atypical genes. The following conclusions can be made from these curves:

1. ShadowCaster identifies with the highest precision the HGT events from medium-far and far donors due to the differences in nucleotide composition content and orthologs. The recommended value of *nu* is 0.4.
2. At the end of the parametric component, close HGT events are complex to classify within the group of atypical genes due to the similarity they share with the recipient sequence. This issue can be solved by incrementing the *nu* value but it is important to emphasize that will also increase the false positive rate (FPR).
3. True and False Positive Rates did not change significantly with the increase of the number of proteomes/species in the phylogenetic shadow. The recommended starting number of proteomes is 15 since a small drop of the FPR was observed for the first time at this value. However, for extensive analyses in real datasets where the origin of the HGT is unknow, the number of proteomes could be increased up to 25. Considering that this parameter does not significantly affect the TPR and FPR, adding relevant information to the phylogenetic shadow tend to improve the quality of HGT detections. Please, see an example of ShadowCaster′s predictions belonging to its parametric and phylogenetic component on the real dataset used in the next section at https://github.com/dani2s/ShadowCaster_testData.

### 3.2. Performance on a Real Dataset. Comparison with the Most Popular State-Of-The-Art Computational Tools

To show the purpose of using a hybrid approach in the detection of HGT events, we validate ShadowCaster with a real genome, *Rhodanobacter denitrificans* 2APBS1 (NC_020541.1), retrieved from Hemme et al. [3]. *Rhodanobacter denitrificans* sp. belong to the Gamma-proteobacteria (g-proteobacteria) class that is populated by several genera resistant to environmental harsh conditions, e.g., low pH, high temperatures and concentrations of sulfur, nitrate and metals. In particular, *Rhodanobacter* populations are frequently found within niches with low pH and high levels of nitrate and heavy metals [3]. Hemme et al. analyzed fifty-one genes related to heavy metal resistance in the metagenome of *Rhodanobacter* populations living in contaminated-groundwater. A total of 39 genes were identified as HGT genes by at least one bioinformatic tool. We compare our results to the two best performing tools applied on their work, DarkHorse and AlienHunter, and also to another of the state-of-the art methods based on blast searches, HGTector.

As we ignore the origin of the HGT events, we took the best values obtained from the experiments with the three simulated datasets to run ShadowCaster, the *nu* parameter was set to 0.4 and the number of proteomes to build the phylogenetic shadow to 25. With the aim of comparison, DarkHorse and AlienHunter were run in the same way as mentioned in Hemme′s methods [3]. For more details about the parameter settings used to run the three tools, see Table S1 of Supplementary File S1. Furthermore, the elapsed time during the HGT detections in *Rhodanobacter denitrificans* was estimated for each software, considering as putative donors a non-redundant subset of genomes/taxa from the NCBI (File S2). Such genome-wide detections were performed by using a workstation (Intel(R) Xeon(R) CPU E5-2640 v2 @ 2.00GHz) that uses 16 threads with a RAM memory of 64 GB and Ubuntu 18.04.4 LTS as Operating System.

**Figure 3:**
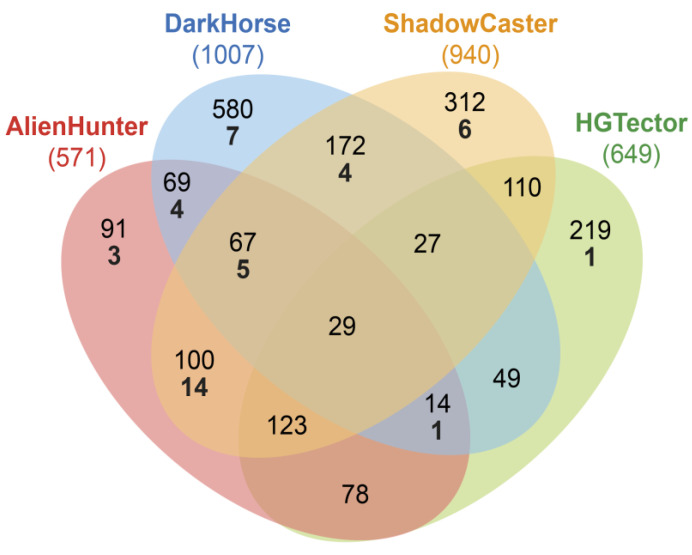
Venn diagram illustrating the HGT events detected by three of the state-of-the-art computational tools (AlienHunter, DarkHorse and HGTector), and by the presented methodology ShadowCaster in the genome of *Rhodanobacter denitrificans* 2APBS1. HGT predictions performed by each tool is framed inside a coloured ellipse. All HGT detections are shown for each tool (black numbers inside each ellipse): AlienHunter (571), DarkHorse (1007), HGTector (649) and ShadowCaster (940) while HGT events related to heavy metal resistance are labelled in bold numbers: AlienHunter (27), DarkHorse (21), HGTector (2) and ShadowCaster (29). Elapsed time during the HGT detections by AlienHunter (31 min 43 s), DarkHorse (93 min 57 s), HGTector (66 min 03 s) and ShadowCaster (103 min 02 s).

HGT detections of ShadowCaster and of the other methods are summarized in Figure 3. The running-time for each method is shown in the figure′s legend. The use of our hybrid approach allows identifying a consensus of genes that are separately identified by the other methods. ShadowCaster and AlienHunter were the two best tools to detect HGT genes associated with metal resistance.

**Figure 4:**
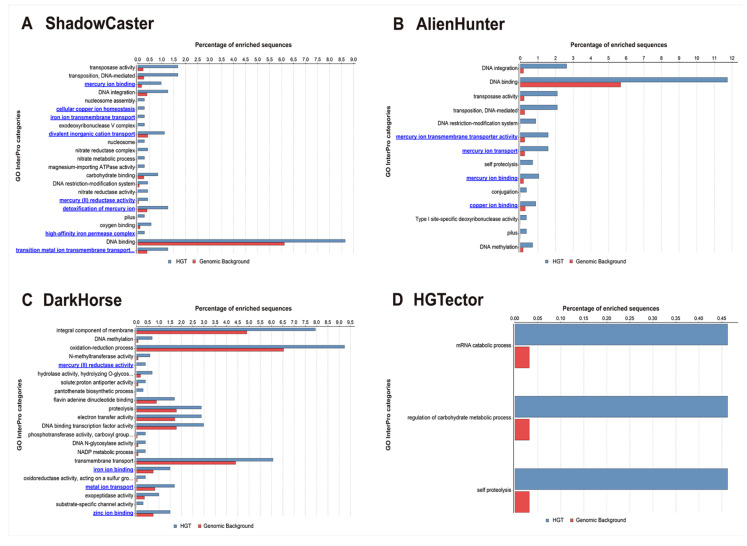
Gene Ontology (GO) Enrichment Analysis. Distribution of GO-InterPro terms exhibiting statistical significance difference (Fisher Exact Test, filtering p-values for multiple testing using False Discovery Rate) for all HGT detections performed by ShadowCaster (**A**), AlienHunter (**B**), DarkHorse (**C**) and HGTector (**D**) in the genome of *Rhodanobacter denitrificans* 2APBS1. InterPro categories highlighted in blue are explicitly related to heavy-metal metabolism. The analysis was conducted using the Blast2GO PRO version.

In prokaryotes, genes acquired horizontally typically tend to code enzymes that are responsible for the growth of metabolic networks [24]. Because this dataset represents a microbial population with extreme exposure to heavy metal contamination, we wanted to understand the contribution degree of the genes detected as HGT to the metabolic pathways for heavy metals. The analysis of Gene Ontology (GO) terms (Figure 4) shows that the genes predicted by ShadowCaster enrich more for enzymatic activities/systems related to heavy metals metabolism (highlighted in blue) than those of AlienHunter, DarkHorse and HGTector. These results confirm the benefits of using a hybrid approach in the detection of HGT events. 

## 4. Conclusions

We presented a new software called ShadowCaster, aimed to improve the detection quality of HGT events in prokaryotes by reducing the number of false positives and the frequently disagreements between the predictions made by parametric methods and by those with implicit phylogenetic models. ShadowCaster is a hybrid approach that sequentially combines a parametric method consisting in One-class SVM classifier trained with two types of compositional features (*k-*mers and codon usage) under the shadow of an implicit phylogenetic model built on the basis that the number of orthologs shared between two species is a proxy of the phylogenetic distance. Thus, it implements an evolutionary model to calculate a Bayesian likelihood for each predicted atypical gene with an unusual sequence composition according to the host genome background in order to detect “true” HGT events in prokaryotes. The software successfully predicted close and distant HGT events in both artificially modified and unaltered bacterial genomes. Its predictions showed the highest agreement with those obtained by state-of-the-art HGT predictive tools, solving, to some extent, the issue addressed by Dessimoz et al. [4] about how to combine different methods or analyzing their predictions without affecting false positive rates.

ShadowCaster can be found at https://github.com/dani2s/ShadowCaster as an open-source software under the GPLv3 license. Source code is hosted at and documentation at https://shadowcaster.readthedocs.io/en/latest/.

## Figures and Tables

**Table 1:** State-of-the-art computational approaches for horizontal gene transfer detection with emphasis in prokaryotic genomes.

Classification	Implementation	Methodological Highlights	Application Domain	Reference
**Parametric methods**				
**Nucleotide composition**	Alien Hunter (http://www.sanger.ac.uk/science/tools/alien-hunter)	Uses Interpolated Variable Order Motifs (IVOMs) coupled to a Hidden Markov Model (HMM) to detect alien (atypical genes).	bacterial genomes	[16]
	No implementation available	Detects atypical genes based on *k*-mer (*k* = 8) frequencies using a one-class support vector machine (SVM).	viral, archaeal and bacterial genomes	[17]
	No implementation available	Combines two compositional features using a Kullback–Leibler divergence metric to improve the detection of atypical genes.	artificial genomes	[6]
	GOHTAM (http://gohtam.rpbs.univ-paris-diderot.fr/)	Uses a Jensen-Shannon divergence metric from window or gene-based signature data to detect atypical genes.	prokaryotic and eukaryotic genomes	[18]
	No implementation available	Detects atypical genes based on the selection of nine compositional features using a SVM.	bacterial genomes	[19]
**Nucleotide composition plus information from the genomic context**	No implementation available	Implements a multiple-threshold approach to detect atypical genes from compositional features and genomic context information to reduce the chance of misclassification.	artificial genomes	[20]
**Implicit phylogenetic methods**				
**Phyletic distributions based on BLAST searches**	DarkHorse (http://darkhorse.ucsd.edu/)	Calculates a lineage probability index from BLAST searches to predict atypical genes.	prokaryotic and eukaryotic genomes.	[13]
	HGTFinder (http://cys.bios.niu.edu/HGTFinder/HGTFinder.tar.gz)	Calculates a horizontal transfer index from BLAST searches to predict atypical genes.	prokaryotic and eukaryotic genomes.	[12]
	HGTector (https://github.com/DittmarLab/HGTector)	Establishes statistical thresholds to detect genes that do not adhere to a priori defined hierarchical evolutionary categories inferred from BLAST searches.	artificial, prokaryotic and eukaryotic genomes.	[14]
**Hybrid methods**				
**Nucleotide composition complemented with an implicit phylogenetic model**	ShadowCaster	See further	prokaryotic genomes	This work

## Supplementary Materials

The following are available online at https://www.mdpi.com/2073-4425/11/7/756/s1. File S1 contains Table S1: Settings used for running state-of-art methods and output processing during the detection of HGT events in *R. denitrificans 2APBS1*; File S2: List of genomes/taxa considered for HGT detections at multiple genome-scale.
